# Radionuclides in Diagnostics and Therapy of Malignant Tumors: New Development

**DOI:** 10.3390/cancers14020297

**Published:** 2022-01-07

**Authors:** Vladimir Tolmachev, Anzhelika Vorobyeva

**Affiliations:** 1 Department of Immunology, Genetics and Pathology, Uppsala University, 751 85 Uppsala, Sweden; vladimir.tolmachev@igp.uu.se; 2 Research Centrum for Oncotheranostics, Research School of Chemistry and Applied Biomedical Sciences, Tomsk Polytechnic University, 634050 Tomsk, Russia

The interest in using targeted radiopharmaceuticals in nuclear oncology has increased in recent years and continues to grow. Radiopharmaceuticals are designed to precisely deliver a radionuclide to cancer-associated molecular targets with either diagnostic or therapeutic purposes. Positron-emitting radionuclides are used for positron emission tomography (PET) imaging, while gamma-emitters are best suited for single-photon emission computed tomography (SPECT) imaging. Radionuclide molecular imaging allows for the whole-body evaluation of cancer-associated targets expressed in real time and therefore aims to make cancer treatment personalized and more effective. As a noninvasive procedure that can be performed repeatedly, it can overcome some of the limitations of conventional biopsy-based diagnostics, which include: a limited number of samples, the inability to sample certain locations, the heterogeneity of target expression as well as the changes in expression over time [1]. By providing additional information, radionuclide molecular imaging helps clinicians in the diagnosis and staging of cancer, as well as in the development of treatment strategies and in the monitoring of target expression in response to treatment [2]. For therapeutic purposes, emitters of alpha and beta particles, as well as Auger electrons, can be utilized. Radionuclide therapy is not subject to multidrug resistance, which is often the case for chemotherapy. A cross-fire effect is also helpful in overcoming an intratumoral heterogeneity of a target expression. Recent approval of the peptide-based agents [^68^Ga]Ga-DOTATATE, [^177^Lu]Lu-DOTATATE and [^68^Ga]Ga-DOTATOC by the FDA (U.S. Food and Drug Administration) marked a breakthrough in targeted radionuclide imaging and therapy and has provided impetus for further development in nuclear oncology. Such developments have included: the discovery of novel cancer-specific molecular targets and investigations of novel classes of targeting agents, as well as the design of targeted radiopharmaceuticals, which has included aspects of radiolabeling chemistry, structure–property relationship, and modifications leading to the desired biological behavior in vivo.

This Special Issue aims to present a cross-section of the state of the art in this research area, highlighting its exciting development.

For a long time, targeting was mainly focused on malignant cells. However, recent research indicates the essential role of the tumor microenvironment (tumor vasculature, fibroblasts, extracellular matrix components, tumor-associated macrophages, neuroendocrine, and adipose cells) in the proliferation and invasion of cancer and which might constitute up to 50% of the tumor volume. A review by Koustoulidou and co-workers is dedicated to radionuclide targeting of cancer-associated fibroblasts (CAFs) [3]. The authors provide a thorough account of the basic biology and role of CAFs in tumor development and metastasis and describe CAF-directed radiolabeled compounds with an apparent potential for radionuclide imaging and therapy. In particular, derivatives of the small-molecule fibroblast activated protein inhibitor (FAPI) are highlighted as possible targeting agents.

Success in radionuclide imaging largely depends on the capacity to form a stable conjugation between the radionuclide and the targeting moieties. For more than a decade, the stable labeling of antibodies with the long-lived positron emitter ^89^Zr (T_1/2_ = 78.4 h) was a challenge for experts in chelator development and radiochemistry. This nuclide is essentially the only option for immunoPET when using full-length monoclonal antibodies. ImmunoPET is helpful in the development of targeted therapeutics, by elucidating the pharmacokinetics and target engagement [4]. For a long time, desferrioxamine (DFO) was used as the chelator of choice for labeling antibodies with ^89^Zr. However, complexes of ^89^Zr with this hexadentate chelator are not sufficiently stable, and any released radionuclide is then accumulated in the bones. A review by Feiner and co-workers [5] describes the progress in the development of the next generation of chelators for zirconium. The authors emphasize the potential of new octadentate hydroxamate-based chelators, which outperform DFO in radiolabeling kinetics and/or complex stability.

Historically, radionuclide molecular imaging utilized monoclonal antibodies as targeting vectors. However, an accumulation of scientific information shows that smaller proteins and peptides are in fact more promising for this purpose. Such imaging probes rapidly extravasate and localize in tumors, and any unbound imaging probes are promptly eliminated from the blood via the kidneys [6]. Consequently, they provide high-contrast imaging within a few hours compared to that of several days when large antibodies are used instead. Still, more comprehensive development regarding all aspects of molecular design is required to utilize the full potential of such targeting agents.

Single domain antibodies (sdAb or VHH) are the smallest immunoglobulin-based targeted probes. Their molecular weight (15 kDa) is ten-fold lower than the weight of IgG antibodies (150 kDa), thus offering the advantage of much higher extravasation rates. Renard and co-workers suggested a technology platform, which enables the simultaneous site-specific coupling of both a diethylenetriaminepentaacetate (DTPA) chelator for radionuclide labeling and a photosensitizer (IRDye700DX) to the epidermal growth factor receptor (EGFR)-binding VHH called 7D12 [7]. The authors used a methodology based on the interaction of dichlorotetrazine with bicyclo[6.1.0]nonyne. In vitro, the radiolabeled conjugate, [^111^In]In-DTPA-IRDye700DX-7D12, bound specifically to EGFR-expressing cells and was cytotoxic in combination with illumination. The accumulation of DTPA-IRDye700DX-7D12 in EGFR-expressing A431 xenografts was demonstrated using SPECT and fluorescence imaging. These types of probes could be utilized for image guided photodynamic therapy.

Engineered scaffold proteins (ESPs) might offer an alternative to immunoglobulins for the development of imaging agents. ESPs are small (4–16 kDa) and can be produced in prokaryotic hosts, which reduces manufacturing costs. Due to their high refolding capacity, labeling of ESPs can be performed in harsh conditions (e.g., heating, acidic or basic pH), which gives more freedom to select an appropriate labeling methodology. Rinne and co-workers [8] evaluated the use of a 68Ga-labeled ESP, affibody molecule [^68^Ga]Ga-(HE)_3_-ZHER3:08698-NODAGA, for the PET imaging of xenografts expressing human epidermal growth factor type 3 (HER3). [^68^Ga]Ga-(HE)^3^-ZHER3:08698-NODAGA was compared with the 89Zr-labeled antibody seribantumab and its fragment, seribantumab-F(ab’)2. The optimal imaging time for [^68^Ga]Ga-(HE)_3_-ZHER3:08698-NODAGA was 3 h, while the optimal contrast provided by [^89^Zr]Zr-DFO-seribantumab and [^89^Zr]Zr-DFO-seribantumab-F(ab’)2 was achieved at 96 h and 48 h pi, respectively. Importantly, the affibody molecules also provided the best imaging contrast.

While antibody-based imaging agents have been studied over several decades, much less is known about ESPs. Multiple structure–property relationship studies are necessary to find an optimal molecular design for ESP-based imaging probes. Deyev and co-workers [9] investigated how both position and composition of the radioactive label influences targeting properties of the designed ankyrin repeat protein (DARPin) Ec1, which binds to the epithelial cell adhesion molecule (EpCAM). Ec1 was labeled with the radiometals ^68^Ga, ^57^Co and ^111^In (using DOTA as the chelator) and with 125I, using ((4-hydroxyphenyl)-ethyl)maleimide (HPEM) as the linker. The authors demonstrated that, for site-specific labeling, C-terminal positioning of all the above mentioned labels resulted in better tumor-to-organ ratios compared to N-terminal positioning. It was found that ^111^In provided the best contrast and, consequently, sensitivity for imaging. The authors concluded that both the label’s position and composition are important in achieving sensitive radionuclide diagnostics when using DARPins.

A small size is also an advantage for short radiopeptide ligands that bind receptors that are overexpressed in malignant tumors. Gastrin-releasing peptide receptor (GRPR), which is overexpressed in breast and prostate cancers, is a target for radiolabeled analogues of the peptide bombesin. The recent implementation of antagonistic bombesin analogues has mitigated the adverse effects, which were previously observed after injection with agonistic analogues. Nock and co-workers [10] have demonstrated that incorporation of the unnatural amino acid sarcosine instead of Gly^11^ in the GRPR antagonist demobesin appreciably improved in vivo stability of this peptide. The tracer, [^99m^Tc]Tc-DB15, demonstrated excellent targeting of GRPR-expressing human breast and prostate cancer xenografts in mice. Moreover, a proof-of-principle study in two patients with breast cancer has demonstrated that injections of [^99m^Tc]Tc-DB15 do not elicit adverse effects, and the tracer enables the visualization of metastases.

Progress in targeted radionuclide therapy might be achieved with the implementation of advanced targeting vectors and the use of optimal radionuclides for each given application.

Targeting of prostate specific membrane antigen (PSMA) with small molecule ligands labeled with different cytotoxic radionuclides demonstrated apparent clinical potential in the treatment of castration-resistant prostate cancer. Bernhardt and co-workers [11] performed a theoretical dosimetry modelling to determine absorbed doses that would be required for metastatic control, if radionuclides ^90^Y, ^131^I, ^177^Lu, ^225^Ac, and ^161^Tb were used for PSMA-targeted therapy. The calculations demonstrated that the required doses are dependent on the metastasis pattern. However, an important result was that the low energy beta emitter 161Tb, which also emits abundant short-ranged conversion and Auger electrons, would be very efficient in the treatment of micrometastases. The authors concluded that [^161^Tb]Tb-PSMA ligands would have the highest potential for improving the response rates of advanced metastatic prostate cancers.

Broqueza and co-workers [12] reported the development and initial characterization of human antibodies that bind to the insulin-like growth factor-2 receptor (IGF2R) overexpressed in osteosarcomas (OS). A phage-display library was used to generate three Fab fragments, which had low nanomolar affinities to the IGF2R of human, canine, and murine origin. After labeling with ^225^Ac, these fragments demonstrated the in vitro killing of IGF2R-positive 143B human OS cells in a dose dependent manner. The Fabs were then converted to full-length human IgGs, conjugated with the versatile chelator CHX-A″-DTPA and labeled with ^111^In. MicroSPECT imaging demonstrated that ^111^In-IF3 accumulated in xenografts generated using human and canine patient-derived cells. The authors concluded that the new antibodies are promising candidates for further development of probes for imaging and therapy of osteosarcoma.

Vilhelmsson Timmermand and co-workers [13] evaluated how labeling chemistry influences in vivo targeting when using the hu5A10 antibody. This antibody binds to PSA in prostate cancer tumors but not to PSA in circulation. After binding, hu5A10 is internalized into target cells by a neonatal fc-receptor (FcRn). The same FcRn regulates hepatic sequestering of antibodies from the blood. The authors evaluated how coupling different numbers of versatile DOTA and CHX-A″-DTPA chelators to hu5A10 influenced its targeting affinity for PSA and FcRn as well as its targeting efficacy and homogeneity of tumor distribution once radiolabeled. An increased number of conjugated chelators decreased the affinity of hu5A10 for both PSA and FcRn, but the magnitude of this impact was different for DOTA and CHX-A″-DTPA. Furthermore, an increased number of coupled CHX-A″-DTPA chelators per antibody resulted in decreasing the hepatic uptake of activity and provided a more homogenous distribution of activity in tumors. In an experimental therapy, using mice with prostate cancer xenografts, the hu5A10 provided significantly better tumor control when it carried the maximum number of chelators. Evidently, this study demonstrates the importance of carefully evaluating the impact of labeling chemistry on the targeting properties of antibodies in order to develop successful therapeutic constructs.

The work of Grob and co-workers [14] has been dedicated to further improving analogues of the peptide minigastrin for targeting of cholecystokinin-2 receptor (CCK2R). Overexpression of CCR2R in a number of cancers, for example, in small cell lung and medullary thyroid cancer, has prompted the development of agents for radionuclide therapy, which target this receptor. One of the major issues with the development of minigastrin analogues is their vulnerability to enzymatic biodegradation in vivo. The replacement of amide bonds with isosteric 1,4-disubstituted 1,2,3-triazoles in targeting peptides is a possible way to improve their proteolytic stability. The authors evaluated how the substitution of peptide bonds with 1,2,3-triazoles in PP-F11N, a CCK2R-targeting ^177^Lu-labeled minigastrin analogue, would modify its stability and targeting properties.

Two of the three evaluated variants NMG2 and NMG3, having triazoles between Tyr^12^ and Gly^13^, had significantly increased affinities and internalization rates compared to PP-11FN. In the case of NMG1, with triazoles between Trp^12^ and Nle^15^, both its affinity and internalization rate decreased. The in vivo stability of PP-11FN and NMG3 to membrane-bound peptidases were approximately equal. Experiments with mice, bearing human CCK2R-positive xenografts, demonstrated that ^177^Lu-labeled NMG2 and NMG3 showed improved tumor uptake, 1.8- and 1.9-fold, respectively, compared with ^177^Lu-labeled PP-11FN. Furthermore, the retention of activity in tumors also increased, which would be favorable for therapy applications. The improved tumor uptake and retention are in a good agreement with the in vitro findings of increased affinities and internalization rates for these peptidomimetics. This study demonstrated the potential of the amide-to-triazole substitution in the development of radiopeptide-based agents for radionuclide therapy of malignant tumors.

While ESPs appeared as excellent imaging probes, their use for radionuclide therapy is hampered by a high reabsorption in the proximal tubuli of the kidneys. A proof-of-principle in vivo study demonstrated that the use of peptide nucleic acid (PNA)-mediated pretargeting enabled efficient affibody-based therapy of HER2-expressing xenografts in mice without renal toxicity [15]. Tano and co-workers [16] evaluated the impact of the ^177^Lu-PNA probe length on radionuclide uptake in tumor and normal tissue. The targeting properties of three PNA variants, 9-mer, 12-mer, and 15-mer, were compared. It was found that the affinity of the radiolabeled PNA variants for HER2-expressing cells, which were pretreated with an affibody-PNA primary agent, was not dependent on their lengths. In mice bearing human ovarian cancer xenografts, the initial tumor uptake was 19–24 %ID/g, with a tendency for higher uptake with shorter PNA probes. Shorter PNA probes had lower uptakes in the kidneys, which is a critical organ for affibody-based PNA-mediated pretargeting. According to the authors’ calculations, use of the shortest PNA variant would enable a twofold increase in tumor dose, compared to that of the longest variant, while still keeping the renal dose the same.

In conclusion, this Special Issue shows that there is continual progress in the development of targeted radionuclide imaging and therapy for malignant tumors. This progress includes the development of new targeting agents, such as sdAbs or ESPs, and refinement of the molecular design of already well-established ones, such as monoclonal antibodies and radiopeptides. New nuclides and targeting probes are being investigated for a number of promising molecular targets. Acquiring interdisciplinary competence in this area of research and strengthening collaborations between researchers and clinicians would likely facilitate the translation of promising radiopharmaceuticals from preclinical studies into clinical practice.
